# Cellulolytic and proteolytic ability of bacteria isolated from gastrointestinal tract and composting of a hippopotamus

**DOI:** 10.1186/s13568-016-0188-x

**Published:** 2016-03-01

**Authors:** Geomárcia Feitosa da Cruz Ramos, Patricia Locosque Ramos, Michel Rodrigo Zambrano Passarini, Marghuel A. Vieira Silveira, Débora Noma Okamoto, Lilian Caroline Gonçalves de Oliveira, Larissa Vieira Zezzo, Alyne Marem, Rafael Costa Santos Rocha, João Batista da Cruz, Luiz Juliano, Suzan Pantaroto de Vasconcellos

**Affiliations:** Department of Biological Sciences, Federal University of São Paulo, Rua São Nicolau, 210, Diadema, São Paulo 09913-030 Brazil; São Paulo Zoo Park Foundation, Av. Miguel Estéfano, 4241, São Paulo, São Paulo 04301-905 Brazil; Department of Biophysics, Federal University of São Paulo, Rua 3 de Maio, 100, São Paulo, São Paulo 04044-020 Brazil; Latin American Institute of Life Sciences and Nature, Federal University of Latin American Integration, Av. Tarquínio Joslin dos Santos, 1000, Foz do Iguaçu, Paraná 85870-901 Brazil

**Keywords:** Protease, Cellulase, Screening, Biofuel, *Bacillus*

## Abstract

The bioprospection for cellulase and protease producers is a promise strategy for the discovery of potential biocatalysts for use in hydrolysis of lignocellulosic materials as well as proteic residues. These enzymes can increment and turn viable the production of second generation ethanol from different and alternative sources. In this context, the goal of this study was the investigation of cellulolytic and proteolytic abilities of bacteria isolated from the gastrointestinal tract of a hippopotamus as well as from its composting process. It is important to highlight that hippopotamus gastrointestinal samples were a non-typical sources of efficient hydrolytic bacteria with potential for application in biotechnological industries, like biofuel production. Looking for this, a total of 159 bacteria were isolated, which were submitted to qualitative and quantitative enzymatic assays. Proteolytic analyzes were conducted through the evaluation of fluorescent probes. Qualitative assays for cellulolytic abilities revealed 70 positive hits. After quantitative analyzes, 44 % of these positive hits were selected, but five (5) strains showed cellulolytic activity up to 11,8 FPU/mL. Regarding to proteolytic activities, six (6) strains showed activity above 10 %, which overpassed results described in the literature. Molecular analyzes based on the identification of 16S rDNA, revealed that all the selected bacterial isolates were affiliated to *Bacillus* genus. In summary, these results strongly indicate that the isolated bacteria from a hippopotamus can be a potential source of interesting biocatalysts with cellulolytic and proteolytic activities, with relevance for industrial applications.

## Introduction

Microorganisms represent the major source of genetic diversity on Earth. The prestige of microorganisms is due to their high metabolic versatility, which allows the inference about its potential for biotechnological applications, including enzyme production for industrial and environmental uses. In this sense, composting process developed at São Paulo Zoo Park Foundation (SPZPF) can be considered as an important biodiversity source for biocatalytic applications. This process is powered by vegetable and animal residues (feces, carcasses, food remains), including material from a remnant of the Atlantic Rain Forest, where the park is located.

There are strains or consortia of microorganisms that have demonstrated enzymatic potential to break lignocellulosic materials. These compounds are formed by cellulose (40–50 %), hemicellulose (25–35 %) and lignin (15–20 %) (Ragauskas et al. 2006; Zhang et al. 2006). Cellulases (responsible for cellulose cleavage) and xylanases (action under hemicellulose) are special enzymes for applications in processes for lignocellulosic compound break, like biomass from sugar cane residues, specifically bagasse and its derivatives.

The activities of cellulases according to their classification are as follow: (a) endoglucanases (EC 3.2.14.4 1,4-β-d-glucan-4-glucanohydrolase): promotion of β-1,4 linkage break in amorphous regions, reducing the polymerization degree and producing reduced and non-reduced terminals, exposing microfibrils to other enzymatic attack; (b) cellobiohydrolases (EC 3.2.1.91 1,4-β-d-glucancellobiohydrolases): exoglucanase action in monomers (glucose) or dimers (cellobiose) removing them from terminal portion of the chains; (c) β-glucosidase (EC 3.2.1.21): cellobiose hydrolysis and, in some cases, other small oligosaccharides to glucose; (d) swollenin: action under cellulosic fibers without hydrolytic activity (Aro et al. 2005).

The described activities for the xylanases are as follow: (a) endo-1,4-β-d-xylanases (EC 3.2.1.8): hydrolysis of β-1,4 linkage of xylan molecule, reducing the polymerization degree of the substrate; β-xylosidases (EC 3.2.1.37): small xylo-oligosaccharide hydrolysis to xylose; (c) α-l-arabinofuranosidases (EC 3.2.1.55): hydrolysis of α-l-arabinofuranosyl terminal groups; (d) α-glucoronidases (EC 3.2.1.1): hydrolysis of α-1,2 glycosidic linkages between xylose and glucuronic acid; (e) acetyl xylan esterases (EC 3.1.1.72): hydrolysis of xylose, acetic acid and ferulic acid; (f) feruloyl esterases (EC 3.1.1.73): increment of the hydrolytic enzyme access under hemicellulose fibers, through removing ferulic acid from lateral and other chains (Aro et al. 2005; Vries and Visser 2001). These enzymes can be highly efficient for second generation ethanol production, employing sub-products from traditional process based on sugar cane juice, that are rich in lignocellulosic fibers. For the successful and complete cleavage of this type of biomass, all of the sugars must be converted to ethanol, through pentoses and hexoses fermentation. However, pentose fermentation is a complex technique that are not so well implemented in industrial scale, due to high costs involved in the complete process (Martinez et al. 2008).

Brazilian research projects are being developed applying sugar cane residues as raw material for a profitable method to have second-generation ethanol in high scale. However, this vegetable and its sub-products have levels around 3–4 % of crude proteins in their composition (Lima 2007). That is why the use of enzymes like proteases, as well as biocatalytic processes, can be useful for removing soluble proteins that can be interesting for increment cellulose hydrolysis reactions, aiming ethanol production income.

Ethanol production in Brazil is exclusively developed by first generation techniques, through saccharose fermentation, however, if other vegetable parts from sugarcane could be used for this application, the country would improve its biofuel efficiency and reduce the land area for plant cultivation (Gray et al. 2006). Therefore, the prospection for microorganisms to produce enzymes, that are able to convert all polysaccharides and proteins found in lignocelullosic material to ethanol is of strategical importance.

The present work is inserted as some tool in the search for cellulases and proteases from bacteria collected from unusual sources, including samples from the gastrointestinal tract of a hippopotamus as well as its composting after euthanasia, at São Paulo Zoo Park, Brazil.

## Materials and methods

### Sampling

#### Hippopotamus gastrointestinal samples

A female hippopotamus was submitted to euthanasia at São Paulo Zoo Park Foundation (SPZPF)—Brazil, due to a metastatic cancer. In this context, it was allowed, for UNIFESP research groups, to collect gastrointestinal samples, specifically from the animal stomachs and intestines. The material was preserved in sterile plastic bags and maintained frozen at −20 °C.

#### Hippopotamus composting

**Fig. 1:**
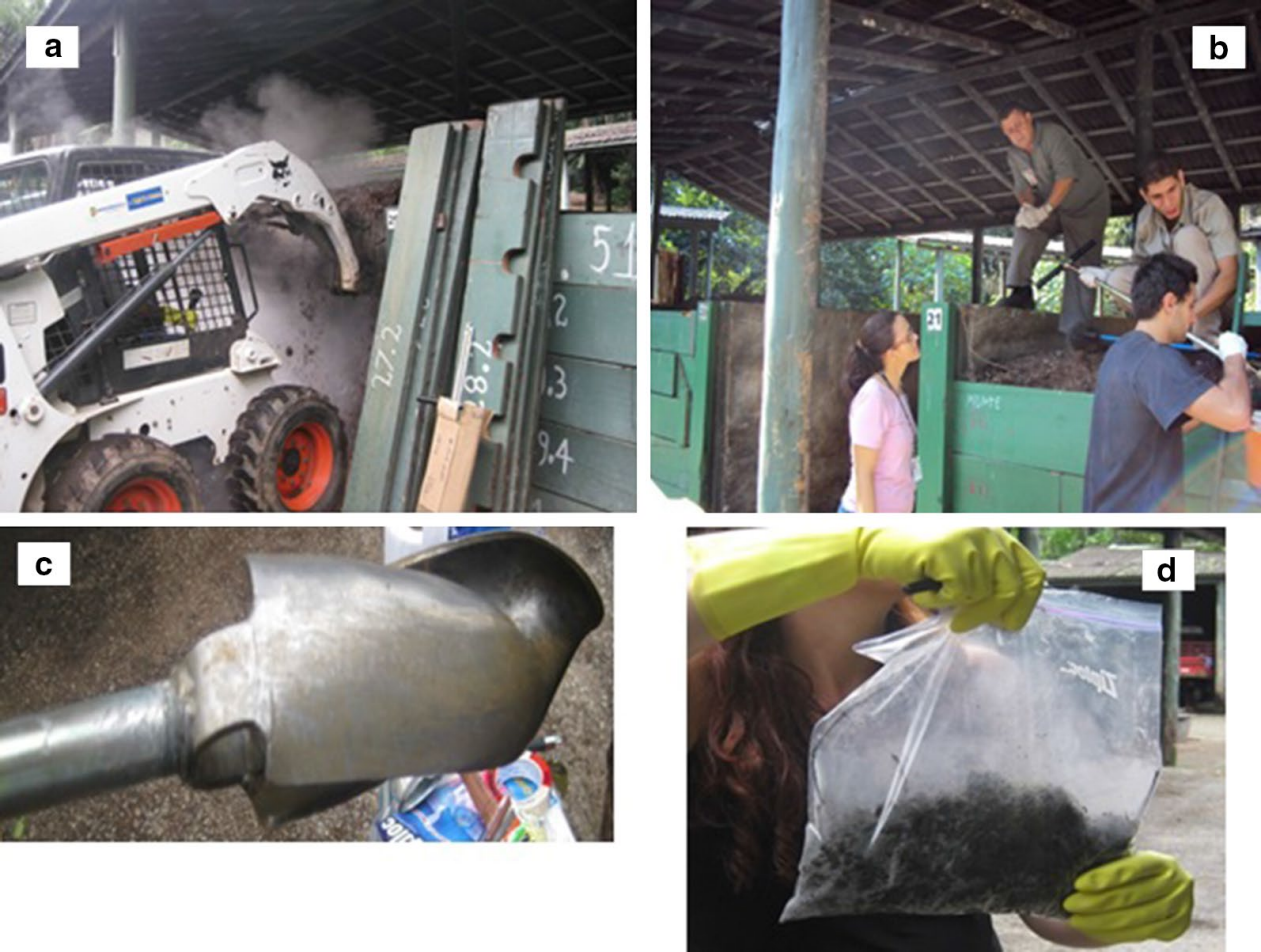
**a** Illustration of the process to construct of a composting pile at São Paulo Zoo Park Foundation; **b** Illustration of technicians sampling material from the composting pile; **c** Adopted instrument that was applied to access all the layers that form the composting pile (Bitencourt et al. 2010); **d** Ready sample for microbiological analyzes

The carcass of the animal was incorporated into the ground vegetable generated at SPZPF (from its gardens, pruning of trees), at the approximate ratio of 30:1 (C:N), as an established procedure adopted by the Environmental Management System at the park. SPZPF has a UNIT for Organic Composting Production (UOCP) and all of the organic residues, that are generated in the maintenance of the Zoo, including animal carcasses, feed residues and manure are composted under controlled conditions (pH, temperature, humidity, aeration). The composting cell of the hippopotamus parts were assembled in August and finished at November, 2011. The cell composting dimension was 2.5 × 2.0 × 1.6 m (length × width × height). After 60 days of the composting process, the material was resolved and the average temperature of the pile was 50 °C. At that time, a sampling from the pile was collected at five different points, according to methods described by Bitencourt et al. (2010), illustrated in Fig. 1.

#### Bacterial cultivation and isolation

Both the collected material from the gastrointestinal tract as well as the composting pile, were prepared using the same protocol. An aliquot of 10 g of sample was mixed in sterile ultrapure water (90 mL), following by vigorous agitation during 5 min and kept in rest during 1 h. Serial dilutions (10^−1^, 10^−3^, 10^−5^) were prepared from these suspensions, which 100 μL of each one, were inoculated in different bacterial culture media, in duplicate. The following media were adopted, according previous experience of the involved research group (data not published): SCA (Starch Casein Agar Himedia); NA (Nutrient Agar, Difco); PCA (Plate Count Agar, Difco); GYA (glucose 20 g/L, Yeast Extract Difco 10 g/L, Bacteriological Agar 20 g/L); GPY Agar (glucose 1 g/L, peptone 0.5 g/L, Yeast Extract Difco 0.1 g/L, Bacteriological Agar 15 g/L); M1 Agar (soluble starch 10 g/L, Yeast Extract Difco 4 g/L, peptone Difco 2 g/L, Bacteriological Agar 15 g/L); TSA (Tryptic Soy Agar Difco); WA (Bacteriological Agar 20 g/L); LB Agar (Luria–Bertani Agar Difco) and ISP2 (Yeast Extract Difco 4 g/L, Malt Extract Himedia 10 g/L, glucose 4 g/L, Bacteriological Agar 20 g/L). All of the media were added by nistatin (25 µL/L), as antifungal agent. The Petri dishes were incubated at 30 and 45 °C, for 72 h. Each macroscopic distinct colony was isolated and streaked in another Petri dish, with the same growth medium for microscopic characterization. All the isolates, after purification, were preserved under −80 °C and maintained at the SPZPF Culture Collection.

The bacterial isolates were denominated as the following acronyms: FPZSP_GITT (SPZPF gastrointestinal samples) and FPZSP_CTT (SPZPF composting process) added by a number for identification.

#### Cellulolytic activity determination

##### Qualitative assays

For the qualitative detection of the cellulolytic potential of the bacterial isolates, a typical methodology, described by Hankin and Anagnostakis (1975) was applied. This method is based on the use of CMC Agar (carboxymethylcellulose) as carbon source in the culture medium and the detection of microbial hydrolytic ability evidenced by Congo Red dye.

##### Quantitative assays

As a form to quantify and confirm the cellulolytic ability detected by the qualitative assays, filter paper was used as substrate, applying an adaptation of the methodology described by Xiao et al. (2004). For this purpose, previously selected bacteria were pre-cultured at Mandels medium (Mandels and Sternberg 1976), with filter paper disks (6 × 6 mm) as substrate, in deep well microplates, in triplicate. The microplates were incubated in a rotational agitator at 150 rpm, 30 °C, during 72 h. After this period, the microplates were centrifuged at 3000*g*, 15 min, at 4 °C. A volume of 32 uL of each supernatant was transferred to another deep well microplate, containing 64 uL of sodium phosphate buffer at pH 7.4 and sodium acetate buffer pH 4.8. Each well was added by another filter paper disk. The reactions were incubated in water bath at 50 °C during 60 min. After this period, 100 uL of Sumner reagent was added, following by incubation at 95 °C, during 5 min. The reactions were transferred to ice bath and 200 uL of each one was transferred to 96 well microplates for absorbance measurements at λ 540 nm. A strain of *Trichoderma reesei* RUT C30 (Borin et al. 2015) (kindly provided by CTBE Brazil—National Laboratory of Bioethanol Science and Technology) was adopted as positive control. Standard curves using glucose as reagent, under the two evaluated pH, were constructed for calculation of the filter paper units (FPU), as well as quantification of the cellulolytic complex activity.

##### Proteolytic activity determination

As a form to complement the enzymatic potential and application of the positive hits obtained from cellulolytic assays, the proteolytic ability was also investigated, through the methodology described by Oliveira et al. (2012). This is a high throughput screening method, based on the use of fluorescence resonance energy transfer (FRET) peptide library probe, under three different pHs (4.0, 7.0 and 9.0). The hydrolysis of the FRET peptides was quantified using a microplate spectrofluorimeter by measuring the fluorescence of Abz (ortho-aminobenzoic acid) as fluorescence donor at λ_ex_ 460 nm, following emission at λ_em_ 360 nm, as well as Q-EDDnp (glutamine-[N-(2,4-dinitrophenyl)-ethylenediamine] as fluorescence acceptor.

#### Identification of the cellulolytic and proteolytic bacteria

##### DNA extraction

Selected bacteria were pre-cultured in Luria–Bertani broth (3 mL), during 24 h at 30 °C, under 150 rpm. DNA extraction was conducted by the use of the commercial kit Wizard Genomic DNA Purification^*®*^, according to the manufacturer’s recommendations. DNA integrity was checked by electrophoresis in 1 % agarose gel and quantified on a NanoVue spectrophotometer (GE Healthcare).

##### 16S rDNA amplification

Amplification analysis of the 16S rRNA gene were developed by polymerase chain reactions (PCR), using 27f and 1401r (Lane 1991) as primers at the following conditions: DNA 6.5 µL, *Taq* Buffer 5 µL; MgCl_2_ (2.5 nM) 1.5 µL, dNTP’s (2.5 nM) 0.4 µL, primers (20 µM) 1 µL, Taq DNA polymerase (Invitrogen) 0.4 U/µL. The PCR amplifications were done using 40 cycles of 2 min at 94 °C, 1 min at 50 °C and 3 min at 72 °C, in a Veriti Applied Biosystems ABI thermal cycler. PCR products (2 µL) were quantified at NanoVue spectrophotometer (GE Healthcare).

##### Sequencing and identification analysis of the bacterial isolates

Amplified products were purified with GFX PCR DNA and Gel Band Purification Kit (GE Healthcare) prior to sequencing. The 16S rRNA gene sequences were sequenced in an automated Genetic Analyzer 3500 sequencer (Applied Biosystems), according to the manufacturer’s recommendations. For these analyzes, purified PCR products (5 µL) were added to BigDye v.3.1 (Applied Biosystems) (4 µL) and sequencing primers (0.5 µL each one). The following primers were adopted 1401r (5´ CGG TGT GTA CAA GAC CC 3´) and 782r (5´ ACC AGG GTA TCT AAT CCT GT 3´). Sequencing program consisted in 25 cycles at 95 °C (20 s), 50 °C (15 s) and 60 °C (60 s).

##### Phylogenetic analyzes

All the phylogenetic analyzes were developed using Mega 4.1 program (Molecular Evolutionary Genetics Analysis). Partial 16S rRNA gene sequences obtained from the pure cultures using the described primers were assembled in a contiguous sequence using the Chromas Pro Current Version 1:34 software. Identification was achieved by comparing the contiguous 16S rRNA gene sequences (~1000 bp in length) generated for isolates with 16S rRNA sequence data from reference and type strains, available at the public databases Genbank (http://www.ncbi.nem.nih.gov) and RDP (Ribosomal Database Project, Wisconsin, USA, http://www.cme.msu.edu/RDP/html/index.html), by using BLASTN and RDP sequence match routines. The sequences were aligned using Clustal W (Thompson et al. 1994) and analyzed using Neighbour-Joining method and *p* distance model. phylogenetic trees were constructed by parsimonium maximum and Neighbour-Joining with bootstrap test in 1000 replicates.

The nucleotide sequences determined in this study for the bacterial isolates were deposited at the Genbank database under the accession numbers: KT884746-KT884759 (Table 1).

**Table 1:** Genbank access of nucleotide sequences and INCQS microbial collection deposit numbers of the selected FPZSP bacterial isolates

Isolates	Genbank access	INCQS deposit number
FPZSP 617	KT884746	P4887
FPZSP 621	KT884747	P4888
FPZSP 630	KT884748	P4889
FPZSP 631	KT884749	P4890
FPZSP 633	KT884750	P4891
FPZSP 636	KT884751	P4892
FPZSP 662	KT884753	P4894
FPZSP 663	KT884754	P4895
FPZSP 696	KT884759	P4900

## Results

### Bacterial isolation

**Fig. 2:**
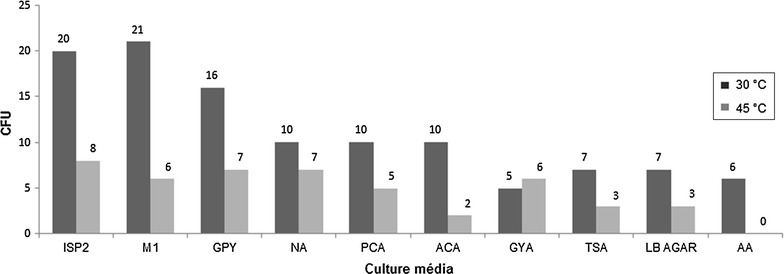
Abundance of bacterial isolates in CFU (colony-forming unit) at different culture media, when incubated at two temperatures: 30 and 45 °C

A total of 159 macroscopically distinct bacterial colonies were isolated, which 62 were from the gastrointestinal samples and 97 from the hippopotamus composting pile. Different culture media and temperature incubation were applied and Fig. 2 shows the results.

### Qualitative screenings for detection of bacterial cellulolytic activity

All the 159 isolated bacteria were screened through qualitative analysis based on the hydrolysis of CMC evidenced by Congo Red dye. It was possible to have 70 positive hits, 40 % of the total screened.

According to the literature (Furlaneto 1989; Bortolazzo 2011), it is possible to establish the enzymatic index value (E.I), as a form to detect cellulolytic strains in potential. This enzymatic index is based on the value of the diameter (cm) of CMC hydrolysis area (Ø h), which is divided by the diameter value (cm) of the bacterial colony (Ø c) (E.I = Ø h/Ø c).

**Fig. 3:**
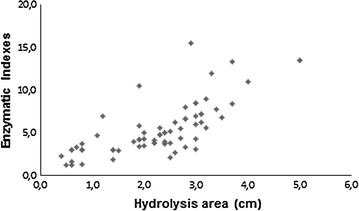
Representation using dispersion curves of enzymatic indexes obtained by 70 selected bacteria

The Fig. 3 illustrates the E.I values obtained from the 70 selected strains, represented as a dispersion curve.

### Bacterial cellulolytic activity quantification

All the 70 positive hits detected in the qualitative screening were evaluated about their cellulolytic abilities, quantifying the acting enzymatic complex by the use of the well-known filter paper methodology, with adaptations of the assays described by Coward-Kelly et al. (2003) and Xiao et al. (2004).

**Fig. 4:**
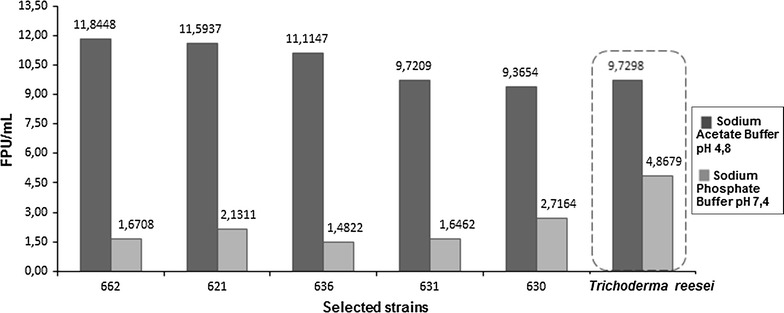
Cellulolytic activity represented as Filter Paper Units (FPU/mL) of five (5) selected strains, under pH 4.8 and pH 7.4, compared to *T. reesei* (positive control)

From the 70 isolates, five (5) strains showed results that were considered as highly active, if compared to the adopted positive control *T. reesei*. Figure 4 ilustrates the obtained data from these five (5) isolates, at the two evaluated pH (4.8 and 7.4).

### Proteolytic activity detection

The 70 selected bacteria on the preliminary cellulolytic screenings were also investigated about their proteolytic ability. This analysis was developed aiming to find proteases for the improvement of bioethanol production, when raw materials rich in protein contents are applied in the process. As a methodology approach, the routine described by Oliveira et al. (2012) was adopted, which is based on the use of FRET peptide probes for bacterial protease detection.

**Fig. 5:**
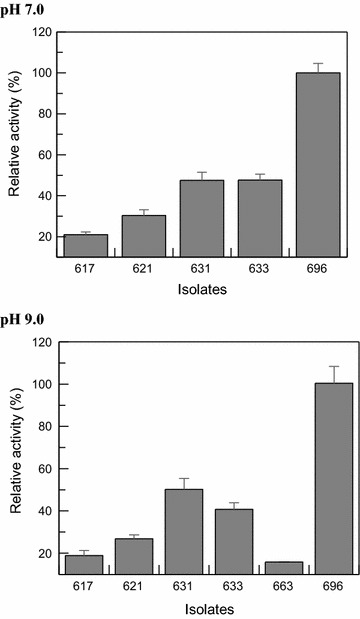
Graphic representation about the proteolytic activity of five (5) bacteria, under pH 7.0 and six (6) isolates under pH 9.0 (cut off 10 %)

Eighteen (18) isolates were detected as positive hits, while 5 (five) were considered potentially actives when evaluated at pH 7.0, and 6 (six) at pH 9.0 (Fig. 5).

### Identification of bacteria with interesting enzymatic potential

#### 16S rDNA analysis

**Fig. 6:**
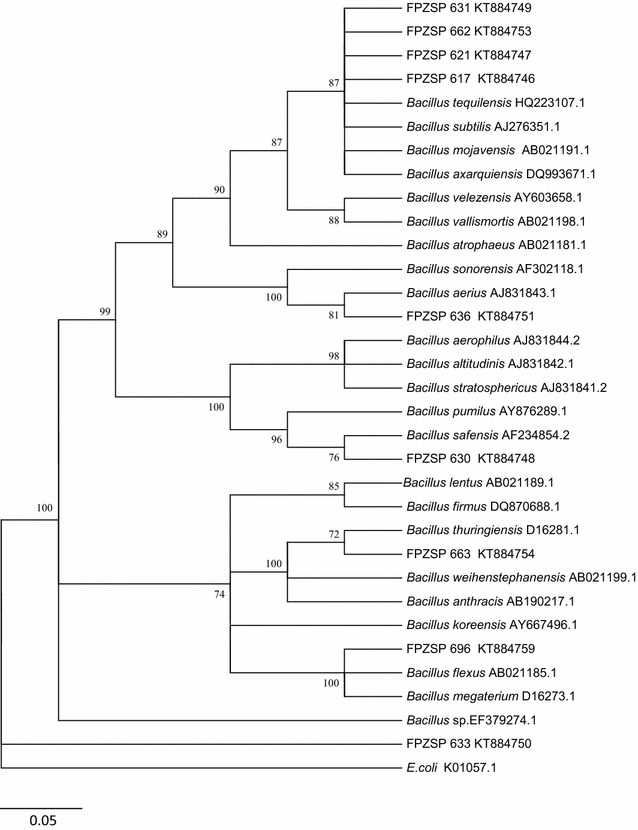
Phylogenetic analysis based on partial 16S rRNA sequences of the FPZSP isolates and related *Bacillus* species. The tree reconstruction was generated from Neighbour-Joining method, following *p* distance model. Bootstrap values (1000 replicate runs,  %) >70 % are listed. GenBank accession numbers are listed after species names. *E. coli* was used as outgroup

Analyzes based on bacterial DNA extraction, as well as PCR technique for 16S rDNA amplification, followed by sequencing and phylogenetic studies allowed the identification of the selected bacteria, about their enzymatic abilities described in this study.All of these selected bacteria were affiliated to *Bacillus* sp. genus. As a form to affiliate these isolates, it was constructed a phylogenetic tree (Fig. 6).

All the bacterial isolates were deposited at INCQS (Culture Collection of the National Institute of Quality Control of Health) (Table 1).

## Discussion

It is estimated that around 10 % of the microorganisms could be already described and identified, as well as only 1 % of them can be cultured at laboratory conditions (Orphan et al. 2000; Burke et al. 2011). To enrich this scenario, a good approach to increment knowledge about biocatalysis mechanisms that are intrinsic to microbial metabolism, could be the use of culture media with different supplements, and prospection of environments that were not or low explored, as a form to reveal the biotechnological potential from microorganisms.

In the present study, it was aimed the access to culturable bacteria, from samples of the gastrointestinal tract of a hippopotamus, as well as from its composting process post-euthanasia. In terms of undescribed and exotic environmental samples, this work is an innovative proposal to show the composting process as adopted at SPZPF, as well as its animal collection as a real source of microbial resources with some enzymatic abilities still not described.

The initial approach of this work was the bacterial isolation from the collected samples. For that purpose, ten (10) culture media formulations were used for microbial recovery. According to the results showed by the Fig. 2, the formulations ISP2 and M1 were more efficient to recovery mesophilic bacteria (41 isolates). Regarding to ISP2, as well as NA and GPY, when the adopted temperature was 45 °C, it was possible to recover 22 bacterial isolates, evidencing them as the preferential media at this condition.

In total, it was possible to recover a total of 159 bacterial isolates, those were screened for their enzymatic potential, specifically for cellulolytic and proteolytic abilities. Preliminary analysis for cellulolytic screenings was based on the use of CMC as substrate, aiming the qualitative detection of bacterial strains with the enzymatic ability. This assay has the positive hits showed as hydrolytic halos that are evidenced by Congo Red dye, which has strong interaction with polysaccharide β-1,4 linkages (Teather and Wood 1982). It constitutes an efficient and quick method for rapid detection of the cellulolytic potential of microorganisms.

As a result, 70 strains were detected as positive hits, but it is important to emphasize that it is a qualitative assay, and many factors including culture medium composition, pH, temperature and colony morphology must be considered for analysis based only in the enzymatic indexes values, since these results are directly related to the microbial growth. In this sense, a high enzymatic index that is obtained for bacteria does not correspond to a fungal strain, since they have strong differences, not only morphological, but also metabolic. This discussion is necessary to clarify the necessity of quantitative methods, like filter paper methodology (Xiao et al. 2004), that allows the stablishment of how much the microbial enzyme is active under the offered substrate.

This could be verified by the results showed by four, that five (5) bacterial isolates had effective cellulolytic ability under filter paper as substrate. It was possible to verify as well, that pH 4.8 was the preferential condition for the enzymatic ability, repeating the same profile showed by the positive control. These results are corroborated by the literature data that also described pH 4.8 as ideal for cellulolytic ability, including activities under sugarcane bagasses and other lignocellulosic sources (Jahangeer et al. 2005; Rabelo 2010).

In the context of ethanol production from alternative sources, as well as the possibilities to agregate technologies in the process, not only cellulase but also the protease activities were screened in the bacteria evaluated in this study. It is well described in the literature that proteases can be added to ethanol process based on the use of starch sources, improving the fermentation step by the cleavage of proteins. Proteases can increase essential yeast nutrients in the form of aminoacids, peptides and free amino nitrogen, as well as promote the hydrolysis of protein substrates, like corn kernel, improving the rate and yield of ethanol. The action of alkaline and acidic proteases from bacterial and fungal strains, including, *Bacillus firmus, Bacillus licheniformis*, *Bacillus subtillis*, *Bacillus cereus* and *Candida albicans* have been reported (Moon and Parulekar 1993; Lantero and Fish 1993; Vidal 2010).

As a form to evaluate the proteolytic abilities of the 70 bacterial strains that were selected for their cellulolytic ability, it was used a high throughput screening methodology based on the use of FRET peptide probe (Oliveira et al. 2012), under three pHs, including neutral, acidic and alkaline conditions. It is important to highlight that the use of FRET probes that are composed by peptide mixtures disposed in a randomic way, can be a facilitator to detect proteolytic abilities when it has a high number of samples, or microorganisms to evaluate. These probes can improve the chances to cleavage peptides in different positions and the results can be easily detected by fluorescence readers.

Eighteen (18) bacterial isolates were detected as positive hits and 5 (five) were considered as potentially interesting at pH 7.0, while 6 (six) at pH 9.0. It is necessary to emphasize that the isolates FPZSP_CTT 621 and 631, those showed proteolytic ability at pH 7.0 and pH 9.0, also presented cellulolytic ability under acidic conditions. This profile comes to the aim of the future application of these bacterial isolates in a real process for ethanol production, because they show a metabolic versatility, which can be improved by future investigations using heterologous systems for expression of the target proteins.

Analyzes to identify the bacterial strains that were selected by investigation of their enzymatic abilities were also developed, allowing their phylogenetic characterization at genus level. In the present study, *Bacillus* sp. constituted the single genus among the bacterial isolates that were selected (Table 1).

Bacteria of the genus *Bacillus*, including known species like *B. subtillis* and *B. pumilus* are well described about their adaptability to many environments, due to their sporulation ability (Keggins et al. 1978; Higgins and Dworkin 2012), which allow them to survive in unconventional conditions, like gastrointestinal tract. Barbosa et al. (2005) also described the presence of *Bacillus* genus in studies about intestinal bacterial microbiota.

In a recent study by Sundberg et al. (2013), representatives of the genus *Bacillus* sp. were also reported as commonly found in the microbial diversity present in composting processes, corroborating to the reported results by the present work.

In this context, the results showed, indicated that the collected samples from the gastrointestinal tract and composting process of a hippopotamus are convenient habitats, when the goal is to search bacterial isolates with enzymatic potential for biotechnological applications.

Summarizing, the investigated samples of bacterial communities cultured from composting of a hippopotamus and the analyzes of their hydrolytic abilities, allowed the isolation of 159 bacteria, of which 70 were positive hits for cellulolytic activity. Among these total, five (5) were potentially interesting to include in cellulosic ethanol processing studies and six (6) showed proteolytic abilities, that may be used to increment biofuel production from protein rich sources.

## References

[CR1] Aro N, Pakula T, Penttila M (2005). Transcriptional regulation of plant cell wall degradation by filamentous fungi. FEMS Microbiol Rev.

[CR2] Barbosa TM, Serra CR, La Ragione RM, Woodward MJ, Henriques AO (2005). Screening for *Bacillus* isolates in the broiler gastrointestinal tract. Appl Environ Microbiol.

[CR3] Bitencourt ALV, Vallim MA, Maia D, Spinelli R, Angeloni R, Principal L, Souza E, Pascon RC (2010). Core sampling test in large-scale compost cells for microorganism isolation. Afr J Microbiol Res.

[CR4] Borin GP, Sanchez CC, Souza AP, Santana ES, Souza AT, Leme AFP, Squina FM, Buckeridge M, Goldman GH, Oliveira JVC (2015). Comparative secretome analysis of *Trichoderma reesei* and *Aspergillus niger* during growth on sugarcane biomass. PLoS One.

[CR5] Bortolazzo NG. Isolamento e seleção de fungos celulolíticos para hidrólise enzimática do bagaço de cana-de-açúcar. Dissertation—Escola Superior de Agricultura “Luiz de Queiroz”, Universidade de São Paulo. 2011.

[CR6] Burke JD, Michael AB, Weintraub N, Charlotte C, Hewins R (2011). Relationship between soil enzyme activities, nutrient cycling and soil fungal, communities in a northern hardwood forest. Soil Biol Biochem.

[CR7] Coward-Kelly G, Aiello-Mazzari C, Kim S, Granda C, Holtzapple M (2003). Suggested improvements to the standard filter paper assay used to measure cellulase activity. Biotechnol Bioeng.

[CR8] Furlaneto MC. Recombinação genética e produção de celulases em *Trichoderma pseudo koningii var.* Dissertation—Escola Superior de Agricultura “Luiz de Queiroz”, Universidade de São Paulo. 1989.

[CR9] Gray KA, Zhao L, Emptage M (2006). Bioethanol. Curr Op. Chem Biol.

[CR10] Hankin L, Anagnostakis SL (1975). The use of solid media for detection of enzyme production by fungi. Mycologia.

[CR11] Higgins D, Dworkin J (2012). Recent progress in *Bacillus**subtillis* sporulation. FEMS Microbiol Rev.

[CR12] Jahangeer S, Kahn N, Jahangeer S, Muhammad S, Shahzad S, Ahmad A, Khan SA (2005). Screening and characterization of fungal cellulases isolated from the native environmental source. Pakistan J Botany.

[CR13] Keggins KM, Lovett PS, Duvall EJ (1978). Molecular cloning of genetically active fragments of Bacillus DNA in *Bacillus subtillis* and properties of the vector plasmid pUB110. Proc Natl Acad Sci USA.

[CR14] Lane DJ, Stackebrandt E, Goodfellow M (1991). 16S/23S rRNA sequencing. Nucleic acid techniques in bacterial systematics.

[CR15] Lantero OJ, Fish JJ. Process for producing ethanol. US patent 5,231,017, 1993.

[CR16] Lima MLP. Cana-de-açúcar na alimentação de bovinos. In: 3^o^ Seminário de Inovações Tecnológicas, Nova Odessa, IZ-SP. National Research Council, 2001 Nutrient Requirements of Dairy Calle;2007. p. 381.

[CR17] Mandels M, Sternberg D (1976). Recent advances in cellulases technology. J Fermentation Technol.

[CR18] Martinez D, Berka RM, Henrissat B, Saloheimo M, Arvas M, Baker SE, Chapman J, Chertkov O, Coutinho PM, Cullen D, Danchin EGJ, Grigoriev IV, Harris P, Jackson M, Kubicek CP, Han CS, Ho I, Larrondo LF, Leon AL, Magnuson JK, Merino S, Misra M, Nelson B, Putnam N, Robbertse B, Salamov AA, Schmoll M, Terry A, Thayer N, Westerholm-Parvinem A, Schoch CL, Yao J, Barabote R, Nelson MA, Detter C, Bruce D, Kuske CR, Xie G, Richardson P, Rokhsar DS, Lucas SM, Rubin EM, Dunn-Coleman N, Ward M, Brettin TS (2008). Genome sequencing and analysis of the biomass-degrading fungus *Trichoderma**reesei* (syn. Hypocrea jecorina). Nature Biotechnol.

[CR19] Moon S, Parulekar SJ (1993). Some observations on protease production in continuous suspension cultures of *Bacillus firmus*. Biotechnol Bioeng.

[CR20] Oliveira LCG, Silva VO, Okamoto DN, Kondo MY, Santos SMB, Hirata IY, Vallim MA, Pascon RC, Gouvea IE, Juliano MA, Juliano L (2012). Internally quenched fluorescent peptide libraries with randomized sequences designed to detect endopeptidases. Anal Biochem.

[CR21] Orphan VJ, Taylor LT, Hafenbradl D, Delong EF (2000). Culture-dependent and culture-independent characterization of microbial assemblages associated with high-temperature petroleum reservoirs. Appl Environ Microbiol.

[CR22] Rabelo SC. Avaliação e otimização de pré-tratamento e hidrólise enzimática do bagaço de cana-de-açúcar para a produção de etanol de segunda geração. PhD Thesis, Universidade Estadual de Campinas. 2010.

[CR23] Ragauskas AJ, Williams CK, Davidson BH, Britovsek G, Cairney J, Eckert CA, Frederick WJ, Hallett JP, Leak DJ, Liotta CL, Mielenz JR, Murphy R, Templer R, Tschaplinski T (2006). The path forward for biofuels and biomaterials. Science.

[CR24] Sundberg C, Yu D, Whittle-Franke I, Kauppi S, Smars S, Insam H, Romantschuk M, Jönsson H (2013). Effects of pH and microbial composition on odour in food waste composting. Waste Manag.

[CR25] Teather RM, Wood PJ (1982). Use of Congo red-polysaccharide interactions in enumeration and chacacterization of cellulolytic bacteria from the bovine rumen. Appl Environ Microbiol.

[CR26] Thompson JD, Higgins DG, Gibson TJ (1994). CLUSTAL W: improving the sensitivity of progressive multiple sequence alignment through sequence weighting, position-specific gap penalties and weight matrix choice. Nucl Ac Res.

[CR27] Vidal B-Jr C. Protease use in ethanol production from dry fractionated corn. PhD Thesis, University of Illinois at Urbana-Champaign. 2010.

[CR28] Vries RP, Visser J (2001). *Aspergillus* enzymes involved in degradation of plant cell wall polysaccharides. Microbiol Mol Biol Rev.

[CR29] Xiao Z, Storms R, Tsang A (2004). Microplate-based filter paper assay to measure total cellulase activity. Biotechnol Bioengin.

[CR30] Zhang PYH, Himmel ME, Mielenz JR (2006). Outlook for celulase improvement: screening and selection strategies. Biotechnol Adv.

